# Statistical Efficiency in Distance Sampling

**DOI:** 10.1371/journal.pone.0149298

**Published:** 2016-03-07

**Authors:** Robert Graham Clark

**Affiliations:** National Institute for Applied Statistics Research Australia (NIASRA), University of Wollongong, Wollongong, Australia; National Institute of Environmental and Health Sciences, UNITED STATES

## Abstract

Distance sampling is a technique for estimating the abundance of animals or other objects in a region, allowing for imperfect detection. This paper evaluates the statistical efficiency of the method when its assumptions are met, both theoretically and by simulation. The theoretical component of the paper is a derivation of the asymptotic variance penalty for the distance sampling estimator arising from uncertainty about the unknown detection parameters. This asymptotic penalty factor is tabulated for several detection functions. It is typically at least 2 but can be much higher, particularly for steeply declining detection rates. The asymptotic result relies on a model which makes the strong assumption that objects are uniformly distributed across the region. The simulation study relaxes this assumption by incorporating over-dispersion when generating object locations. Distance sampling and strip transect estimators are calculated for simulated data, for a variety of overdispersion factors, detection functions, sample sizes and strip widths. The simulation results confirm the theoretical asymptotic penalty in the non-overdispersed case. For a more realistic overdispersion factor of 2, distance sampling estimation outperforms strip transect estimation when a half-normal distance function is correctly assumed, confirming previous literature. When the hazard rate model is correctly assumed, strip transect estimators have lower mean squared error than the usual distance sampling estimator when the strip width is close enough to its optimal value (± 75% when there are 100 detections; ± 50% when there are 200 detections). Whether the ecologist can set the strip width sufficiently accurately will depend on the circumstances of each particular study.

## 1 Introduction

The number of animals in a region is often of ecological importance. This paper considers the distance sampling approach to estimating abundance, in its usual conjunction with line transect sampling. Transect lines are laid down across a region, often parallel and equally spaced but not necessarily so. Observers move along the transects, and record observations of animals (or plants, or groups of animals, or other objects) and their perpendicular distances from the transect. Empirically, more objects are detected near to transect lines than far from them in many studies, suggesting that detectability is a decreasing function of distance. The distance sampling methodology exploits this phenomenon, by modelling the detection rate as a function of distance. The number of detected objects can then be scaled to estimate the abundance *N* allowing for imperfect detection. Provided the assumptions of the method are met, the maximum range can be made fairly large, thereby increasing the sample size, while avoiding or reducing bias due to declining detection rate. Distance sampling is widely used in ecology: a Web of Science search found 276 articles on distance sampling in ecology journals in 2014 alone. The wide range of applications includes wild horses in the Australian Alps [1], large herbivores in South Africa’s Kruger National Park [2] and odonata (dragonflies) in a rainforest locality in Papua New Guinea [3]. For a detailed description of the approach, see [4].

The major assumptions of the method are

1. Detection is perfect at zero distance.
2. The detection function is of known form, with some unknown parameters requiring estimation. Alternatively, model-averaging may be used provided the detection function is assumed to be one of a known set of alternatives.
3. Animals’ distances to the nearest transect line are (at least approximately) uniformly distributed.

It is also assumed that there is no measurement error (for example, false positive detections, or mis-measurement of distances), that there is no movement of objects in response to the observer which could lead to multiple chances of detection, that detection events are independent, that there are sufficient transects for reliable estimation, and that transect lines are a good representation of the study area. This paper will consider the conventional distance sampling (CDS) scenario where there is only one observer. The same methodology can be used with multiple observers by pooling their detections. Mark recapture and mark recapture distance sampling (MRDS) are methods which more fully use data from multiple observers by matching their detections; MRDS, in particular, can be used to relax assumption (i). More recent approaches combine a spatial model with the detection model (see for example [5–7] and [8]). Spatial models allow abundances to be estimated for subregions, and can exploit spatial trends in estimation, however inference may be sensitive to the assumed spatial model which must therefore be carefully constructed. This paper focuses on CDS, as most applications of line transect sampling remain single observer. The use of spatial models in distance sampling is an important advance, but some researchers may decide that the extra effort and complication required to develop an adequate spatial model are not warranted for some studies, particularly when the number of detections is relatively small.

Robustness to the 3 assumptions above is explored in the literature. For example, MRDS can be used to achieve some level of robustness to (i). Assumption (ii) is dealt with by the use of flexible families of detection functions with two or more parameters, and the use of model averaging. [9] argue that (iii) is approximately satisfied provided transect lines are placed randomly or systematically from a random starting point. However, [10] question the uniformity assumption and find that CDS estimators are biased in a design-based framework, that is, under repeated random placement of transect lines. The matter remains in contention [11–13]. [11] suggest an alternative approach where the detection function is estimated from a separate calibration study, and [14] propose estimating detection probabilities using multiple observer data and possibly but not necessarily distance data.

A natural alternative to distance sampling is the simple scaling up of observations in a strip about the transect. When strips are too wide, this strip transect estimator is severely negatively biased due to non-detections, particularly of the more distant animals in the strip. When strips are sufficiently narrow, this bias becomes negligible, but the variance of the estimated abundance becomes large. Distance sampling aims to achieve lower variance by including observations at greater distances, while reducing bias by adjusting for non-detection. But there is a hidden cost—the effect of unknown detection parameters on the precision of the estimated abundance—which reverses at least some of the benefit due to wider strips. This paper illuminates this cost both asymptotically and in small samples. This existence of a penalty due to unknown detection parameters is known, but no asymptotic expression has been derived in the literature, and the penalty has not been quantified except in a simulation of the half-normal and negative exponential detection functions [15]. A number of authors have compared CDS and strip transect estimators in particular applications (e.g. [1, 16]) but typically with only one or two options for strip width. [17] also suggested strip transects as an alternative to CDS but did not make a numerical comparison.

An example of the variability of CDS estimators may be found in [1], which compares strip transect estimates with two different strip widths to CDS estimates with various detection functions, and to mark-recapture and MRDS estimates of abundance. Detections of groups of wild horses are attempted up to 200m from a helicopter traversing 91 parallel transect lines in south-eastern Australia, resulting in 52 group detections (pooled from two observers). Table 1 here reproduces results from page 1145 of [1]. The strip transect estimates of abundance are the number of observed groups within 50m or 200m multiplied by the total area divided by the area lying within either 50m or 200m of a transect line, with no allowance for undercount. The values of the Akaike Information Criterion (AIC) are also shown for each detection model. Model averaged estimates are calculated using weights calculated from the AICs as described in [18]. Mark recapture and MRDS results are not reproduced here as they are outside the scope of the current paper. The CDS estimator shown in Table 1 corresponds to formula (6) in the next section of the current paper.

**Table 1:** Estimates of Density (abundance/area) of Horse Groups obtained from [1]. CV is the estimated coefficient of variation of the estimate, given by the square root of the estimated variance divided by the estimate.

Method	Detection Function	AIC	$\hat{Density}$ $\left(\hat{N}/A\right)$ (groups/km^2^)	$CV\%(\hat{N})$	Strip Width	estimated average detection probability
CDS	neg. exponential	0.00	0.36	24.8	200m	0.50
CDS	uniform (cosine)	0.96	0.28	18.8	200m	0.64
CDS	half-normal	1.35	0.28	19.9	200m	0.64
CDS	hazard rate	1.87	0.36	57.2	200m	0.50
CDS	model-averaged	n/a	0.33	30.3	200m	0.55
Strip	n/a	n/a	0.31	22.1	50m	1.00
Strip	n/a	n/a	0.18	16.1	200m	1.00

Table 1 shows that the CDS abundance estimators have high coefficients of variation (CV), with the two-parameter hazard rate model leading to a much higher CV (about 57%) than the other detection models which are one-parameter (CVs between 19% and 25%). The model-averaged estimator has a CV of 30%. The strip transect estimator using detections up to 200m has much lower CV, but is much lower than the CDS estimators, suggesting that it is negatively biased due to undetected groups. The strip transect estimator based on detections up to 50m is more plausible. It is close to the model-averaged CDS estimator, suggesting that its bias is small, presumably because the detection rate would decline relatively little over this shorter range. Surprisingly, the 0–50m strip transect estimator gives similar CVs to the 0–200m model-averaged CDS method, even though it only uses one quarter of the distance range and 44% of the detected groups. This motivated the research in the current paper on the efficiency of CDS estimators.

Section 2 proves for the first time that the usual CDS estimator of *N* is also a maximum likelihood estimator (MLE) of *N* under a particular model provided the likelihood is approximated using Stirling’s Rule. Its asymptotic variance under the model is derived using this result. The asymptotic variance is identical to that of [19], but the use of the Stirling approximation allows a simpler derivation. The limiting variance of $\hat{N}$ is expressed as the variance when the detection function is known multiplied by a penalty for unknown ***θ***. This asymptotic penalty is tabulated for various detection models. It can be substantial, and for many situations arising in practice is between 2 and 6. Section 3 summarizes a simulation study to evaluate the small sample performance of CDS estimators for various detection functions and strip transect estimators with differing strip widths. Unlike the theoretical result in Section 2, the simulation allows for overdispersion in the counts of animals falling in any given range. Section 4 contains conclusions about the magnitude of the penalty due to unknown detection parameters in CDS, and the relative performance of CDS and strip transect estimators.

## 2 Theoretical results on maximum likelihood estimation of *N*

### 2.1 Notation and background

The aim is to estimate the number of objects, which may be animals, groups of animals or other objects, in a region. Let *N* be the number of objects in a defined study area and *n* be the number of detections made by an observer moving along predefined line transects. Observation may either be one-sided (only objects to the right, or only objects to the left, are observed) or two-sided. Only objects with perpendicular distance up to a pre-chosen limit *w* from a transect line have a chance of being observed (the *covered region*); let *N*_*c*_ be the number of such objects. Two-sided observation is the more common case, but one-sided observation is sometimes necessary, for example if the observer can only see out one side of a vehicle. It is assumed that the probability of observing an object at perpendicular distance *d*_*i*_ from a transect line is *g*(*d*_*i*_, ***θ***) when 0 ≤ *d*_*i*_ ≤ *w*, where ***θ*** is a vector of *p* parameters specifying the function within a family. It is assumed that *g*(0, ***θ***) = 1 and that *g* is a non-increasing function of distance. The Distance software [20], which implements both CDS and MRDS, allows four possible functional forms for *g*(), including the half-normal model, $g\left(d_{i}\right)=\exp(-\frac{d_{i}^{2}}{\theta^{2}})$, and the hazard rate function, $g\left(d_{i}\right)=1-\exp(-{\left(d_{i}/\theta_{1}\right)}^{-\theta_{2}})$. Both of these functions satisfy the *shoulder condition* that *g*′(0) = 0, with the hazard rate model giving greater flexibility in modelling the *shoulder width* (i.e. the range near 0 over which *g* is relatively flat). The use of “robust models”, which have enough flexibility to model a range of typical shapes, is recommended on pages 46–49 of [9], with the hazard rate model given as a particularly useful example.

It is also possible to include other covariates affecting the detectability of objects in the distance function, such as characteristics of the animal or plant (see [21] and [22]).

Let *d*_*i*_, *i* = 1, …, *N*, be the perpendicular distances from the objects to the nearest transect line, and let *δ*_*i*_ = 1 for observed objects and *δ*_*i*_ = 0 for the rest. Under model assumption (iii) stated in the Introduction, *d*_*i*_ are independent and identically distributed uniform *U*(0, *M*) for *i* = 1, …, *N*, where *M* is the maximum possible distance from a transect line. The distribution of *d*_*i*_ given *δ*_*i*_ = 1 is easily derived as

$$\begin{array}{c} g_{cond}\left(d_{i};\theta \right)=g\left(d_{i};\theta \right)/\int_{0}^{w}g\left(u;\theta \right)du \end{array}$$ (1)

Let $\bar{g}(\theta )=w^{-1}\int_{0}^{w}g(u;\theta )du$ be the unconditional probability of detection. (This is the probability that an animal in the covered region is detected, unconditional on its distance, denoted by *P*_*a*_ in equation (2) of [23]. The notation $\bar{g}(\theta )$ is used here to emphasise that it is the mean value of *g*(*d*_*i*_; ***θ***) over *d*_*i*_ ≤ *w*.) The conditional likelihood of ***d***_***s***_ = (*d*_1_, …, *d*_*n*_)^*T*^ given *n* is

$$\begin{array}{c} L_{d}=\prod_{i=1}^{n}g_{cond}\left(d_{i};\theta \right)=\left(\prod_{i=1}^{n}g\left(d_{i};\theta \right)\right){\left(\int_{0}^{w}g\left(u;\theta \right)du\right)}^{-n}=\left(\prod_{i=1}^{n}g\left(d_{i};\theta \right)\right)w^{-n}\bar{g}{\left(\theta \right)}^{-n} \end{array}$$ (2)

and the corresponding conditional log-likelihood given *n* is

$$\begin{array}{c} l_{d}=\sum_{i=1}^{n}\text{log}g\left(d_{i};\theta \right)-n\text{log}\left(\bar{g}\left(\theta \right)\right)-n\log w. \end{array}$$ (3)

The parameters ***θ*** can be obtained by setting the derivative of *l*_*d*_ with respect to ***θ*** to 0. Prior to this, it is convenient to define

$$\begin{array}{c} h\left(u;\theta \right)=\frac{\partial }{\partial \theta }g\left(u;\theta \right) \end{array}$$ (4)

and $\bar{h}(\theta )=w^{-1}\int_{0}^{w}h(u;\theta )du$. Notice that ***h***(*u*; ***θ***) is a p-vector where *p* is the number of parameters in ***θ***. The partial derivative of $\bar{g}(\theta )$ with respect to ***θ*** is $\bar{h}(\theta )$, subject to regularity conditions allowing the derivative operator to be taken within the integral. Setting the derivative of *l*_*d*_ to 0 gives the following estimating equation for ***θ***:

$$\begin{array}{c} 0=\sum_{i=1}^{n}g{\left(d_{i};\theta \right)}^{-1}h\left(d_{i};\theta \right)-n\bar{g}{\left.(\theta \right)}^{-1}\bar{h}\left.(\theta \right) \end{array}$$ (5)

The most commonly used estimator of *N* in CDS is:

$$\begin{array}{c} {\hat{N}}_{CDS}=\frac{n}{P\bar{g}\left({\hat{\theta }}_{CML}\right)}. \end{array}$$ (6)

where *P* is the proportion of the area falling within perpendicular distance of *w* of a transect line, and ${\hat{\theta }}_{CML}$ is the solution to Eq (5) (CML stands for conditional maximum likelihood, as the likelihood *L*_*d*_ in Eq (2) is conditional on *n*). In CDS, the estimation of ***θ*** is model-based and maximises the likelihood conditional on *n*, while the estimation of *N* in Eq (6) is design-based and motivated by the fact that $E\left[n\right]=NP\bar{g}(\theta )$. See equation 1.4 of [9]. See also [23] for a recent discussion of CDS and extensions. The value of *P* is assumed to be known, and is approximated in practice by the total length of all transects multiplied by *w* (and multiplied by 2 for two-sided observation) divided by the region’s area.

Another alternative that has been proposed is maximum likelihood estimation of *N*, which is the minimum variance unbiased estimator for large samples subject to regularity conditions. Section 7.2 of [24] derives the likelihood for *N* and ***θ***. The conditional density of ***d***_***s***_ given *n* is *L*_*d*_ in Eq (2). Under the assumed model, *δ*_*i*_ are independent Bernoulli random variables with expected value $\bar{g}(\theta )$. Hence

$$\begin{array}{c} n∼\text{bin}\left(N,P\bar{g}\left(\theta \right)\right). \end{array}$$ (7)

The likelihood is the product of the probability function of *n* and *L*_*d*_:

$$L=\left(\begin{array}{l} N\\ n \end{array}\right){\left\{P\bar{g}\left(\theta \right)\right\}}^{n}{\left\{1-P\bar{g}\left(\theta \right)\right\}}^{n}L_{d}$$ (8)

$$=\left(\begin{array}{l} N\\ n \end{array}\right){\left\{P\bar{g}\left(\theta \right)\right\}}^{n}{\left\{1-P\bar{g}\left(\theta \right)\right\}}^{N-n}\left(\overset{n}{\underset{i=1}{\prod }}g\left(d_{i};\theta \right)\right)w^{-n}\bar{g}{\left(\theta \right)}^{-n}$$ (9)

$$=\left(\begin{array}{l} N\\ n \end{array}\right)P^{n}w^{-n}{\left\{1-P\bar{g}\left(\theta \right)\right\}}^{N-n}\overset{n}{\underset{i=1}{\prod }}g\left(d_{i};\theta \right)$$ (10)

(See equation 2.33 of [4]). *L* can be maximised with respect to *N* and ***θ*** to obtain a maximum likelihood estimator of *N*. In this approach, both *N* and ***θ*** are treated as unknown parameters. On pages 16–17, [4] recommend against this approach because in practice *n* is likely to be overdispersed and so to have higher variance than implied by Eq (7). For example, this would occur if there were positive correlations between the values of *d*_*i*_. It is worth noting that any such overdispersion is likely to also invalidate Eq (2) and so ${\hat{\theta }}_{CML}$ and ${\hat{N}}_{CDS}$ to some extent, as Eq (2) assumes that *d*_*i*_ are independent conditional on *n*.

When ***θ*** is known, the MLE of *N* is the integer part of $n/(P\bar{g}(\theta ))$ (see pages 17–19 and 138 of [24]). The same reference also shows that if the factorial terms in Eq (10) are approximated using Stirling’s rule and *N* is treated as a continuous parameter, the MLE is then

$$\begin{array}{c} {\hat{N}}_{known\theta }=n/\left(P\bar{g}\left(\theta \right)\right). \end{array}$$ (11)

It is straightforward to derive the variance of ${\hat{N}}_{known\theta }$ using the fact that $n∼\text{bin}(N,P\bar{g}(\theta ))$:

$$\begin{array}{c} \text{var}\left({\hat{N}}_{known\theta }\right)=P^{-2}\bar{g}{\left(\theta \right)}^{-2}\text{var}\left(n\right)=NP^{-1}\bar{g}{\left(\theta \right)}^{-1}\left(1-P\bar{g}\left(\theta \right)\right) \end{array}$$ (12)

When ***θ*** is unknown, [19] note that the MLE of *N* is the integer part of $n/(P\bar{g}(\hat{\theta }))$ where $\hat{\theta }$ is the MLE of these parameters. The next subsection of this paper extends this result by showing that ${\hat{\theta }}_{CML}$ and ${\hat{N}}_{CDS}$ maximise a Stirling approximation to the full likelihood *L*. This enables a theoretical result on the large sample variance of ${\hat{N}}_{CDS}$, albeit under the strong assumption Eq (7). The simulation study in Section 3 relaxes this assumption by including overdispersion.

### 2.2 Derivations of the MLE and its variance based on Stirling’s approximation

The maximum likelihood estimator of *N* under Stirling’s approximation for factorials is derived in this subsection, where the likelihood *L* is given by Eq (10). (Note that in section 3.3.1, [9] consider maximization of *L*_*d*_, not *L*, to estimate ***θ***, where *L*_*d*_, defined in Eq (2), is the conditional likelihood given *n*). Stirling’s rule log(*x*!) ≈ *x*log(*x*) − *x* implies that

$$\text{log}\;\left(\begin{array}{l} N\\ n \end{array}\right)=\text{log}\left\{N!/n!\left(N-n\right)!\right\}$$ (13)

$$\approx N\log\left(N\right)-N-\left(n\log(n)-n\right)-\left\{\left(N-n\right)\log\left(N-n\right)-\left(N-n\right)\right\}$$ (14)

$$\begin{array}{ccc} & = & N\log\left(N\right)-\left(N-n\right)\log\left(N-n\right)-n\log\left(n\right). \end{array}$$ (15)

There is a positive probability that *n* is equal to 0 or *N*, in which case log(*n*) or log(*N* − *n*) are not defined. The limit of the right hand side of Eq (15) can easily be shown to equal 0 in these cases, so the following refinement of Eq (15) is used:

$$\begin{array}{c} \text{log}\;\left(\begin{array}{l} N\\ n \end{array}\right)\approx \left\{\begin{array}{ccc} N\log\left(N\right)-\left(N-n\right)\log\left(N-n\right)-n\log\left(n\right) & \;\text{if}\; & 0<n<N\\ 0 & \;\text{if}\; & n=0\;\text{or}\;n=N \end{array}\right\}. \end{array}$$ (16)

*L* will be maximised with respect to *N* and ***θ*** treating *N* as a continuous parameter. Stirling’s approximation for log(*x*!) is very accurate even for small *x* as long as *x* is at least 2 or 3, and both *N* − *n* and *n* would be well above this in practice. Theorem 1 states the MLEs and the approximate Fisher information.

**Theorem 1** *The model defined by Eqs (1) and (7) is assumed, and it is assumed that the log of the likelihood Eq (10) can be approximated using Eq (16), leading to*

$$\begin{array}{ccc} l & = & N\log\left(N\right)-\left(N-n\right)\log\left(N-n\right)-n\log\left(n\right)+n\log\left(P\right)-n\log\left(w\right)\\ & & +\left(N-n\right)\log\left(1-P\bar{g}\left(\theta \right)\right)+\sum_{i=1}^{n}\log\left(g\left(d_{i};\theta \right)\right) \end{array}$$ (17)

*Let* Ω *be an open set defining the set of feasible values of ***θ***. If there is a unique* ${\hat{\theta }}_{CML}$ *satisfying Eq (5) then it is the maximum likelihood estimator, and the MLE of N on* (0, ∞) *is* ${\hat{N}}_{CDS}$ *in Eq (6).*

*Let D be a random variable with density* $g\left(d\right)/\int_{0}^{w}g\left(u\right)du$ *for* 0 ≤ *d* ≤ *w*, *which is also the distribution of d_i_ conditional on detection. The Fisher Information of* (*N*,***θ***^*T*^)^*T*^ *is approximately equal to*

$$\begin{array}{c} I\left(N,\theta \right)\approx \left[\begin{array}{cc} I_{NN} & I_{N\theta }^{T}\\ I_{N\theta } & I_{\theta \theta } \end{array}\right] \end{array}$$ (18)

*for large N, where*

$$\begin{array}{ccc} I_{NN} & = & N^{-1}{\left(1-P\bar{g}\left(\theta \right)\right)}^{-1}P\bar{g}\left(\theta \right) \end{array}$$ (19)

$$\begin{array}{ccc} I_{\theta \theta } & = & NPw^{-1}\int_{0}^{w}h\left(u;\theta \right)h{\left(u;\theta \right)}^{T}g{\left(u;\theta \right)}^{-1}du+NP\bar{h}\left(\theta \right)\bar{h}{\left(\theta \right)}^{T}{\left(1-P\bar{g}\left(\theta \right)\right)}^{-1} \end{array}$$ (20)

$$\begin{array}{ccc} & = & NP\bar{g}\left(\theta \right)var_{D}\left(h\left(D;\theta \right)/g\left(D;\theta \right)\right)+NP\bar{h}\left(\theta \right)\bar{h}{\left(\theta \right)}^{T}\bar{g}{\left(\theta \right)}^{-1}{\left(1-P\bar{g}\left(\theta \right)\right)}^{-1} \end{array}$$ (21)

$$I_{N\theta }\;={\left(1-P\bar{g}\left(\theta \right)\right)}^{-1}P\bar{h}\left(\theta \right)$$ (22)

Surprisingly, ${\hat{\theta }}_{CML}$ and ${\hat{N}}_{CDS}$ maximise the Stirling approximation to the full likelihood *L*, even though ${\hat{\theta }}_{CML}$ was defined as the maximizer of the conditional likelihood *L*_*d*_ given *n* (not *L*), and ${\hat{N}}_{CDS}$ was motivated by a design-based argument.

Let *V* be the inverse of the Fisher Information matrix in Eq (18). Subject to regularity conditions, maximum likelihood estimators are asymptotically normal with expectation equal to the true parameter values and variance-covariance matrix equal to the inverse of the Fisher Information matrix. Unfortunately these regularity conditions do not hold here, for example condition (M3) of the Central Limit Theorem on pages 499–500 of [25] is not met, because *n* and ***d***_***s***_ are dependent. Moreover, Eq (18) is only the approximate Fisher information based on a Taylor Series expansion, whereas the usual Central Limit Theorem requires the exact Fisher information. [26] uses an alternative method of proof to derive a Central Limit Theorem for the MLE. It is shown in this working paper that *V* is indeed the limiting variance of ${\left(\hat{N},{\hat{\theta }}^{T}\right)}^{T}$. The proof in [26] is essentially a simplified version of the proof of the result in [19] which does not use the Stirling approximation. Theorem 1 is an advance on the result in [19], because it shows that ${\hat{N}}_{CDS}$ is the full maximum likelihood estimator (subject to the Stirling approximation), and also provides a simpler derivation of *V*. Theorem 1 helps explain the finding of [19] that the difference between ${\hat{N}}_{CDS}$ and the exact maximum likelihood estimator (i.e. the MLE when the Stirling approximation is not made) is small asymptotically. The theorem also suggests that there is no need for the calculation of the exact MLE in practice, even when the model assumptions are justified, since ${\hat{N}}_{CDS}$ maximises an excellent approximation to the full likelihood.

It is convenient to express *V* in block form. We will henceforth mostly write *g*(*u*), ***h***(***u***), $\bar{g}$ and $\bar{h}$ for readability, rather than *g*(*u*; ***θ***) etc. Using a standard result on the inverse of a matrix in block form (e.g. 5.16a of [27]), *V* is equal to

$$\begin{array}{c} V=\left[\begin{array}{cc} V_{11} & V_{21}^{T}\\ V_{21} & V_{22} \end{array}\right] \end{array}$$ (23)

where

$$\begin{array}{c} \left.\begin{array}{ccc} V_{22} & = & {\left\{I_{\theta \theta }-I_{N\theta }I_{NN}^{-1}I_{N\theta }^{T}\right\}}^{-1}=N^{-1}P^{-1}\Delta^{-1}{\bar{g}}^{-1}\\ V_{11} & = & I_{\theta \theta }^{-1}+I_{\theta \theta }^{-1}I_{N\theta }^{T}V_{22}I_{N\theta }\\ & = & N\left(1-P\bar{g}\right)P^{-1}{\bar{g}}^{-1}+NP^{-1}{\bar{g}}^{-3}{\bar{h}}^{T}\Delta^{-1}\bar{h}\\ V_{21} & = & V_{21}^{T}=-V_{22}I_{N\theta }I_{NN}^{-1}\\ & = & -{\bar{g}}^{-2}P^{-1}\Delta^{-1}\bar{h} \end{array}\right\} \end{array}$$ (24)

and Δ is the *p* by *p* matrix defined by

$$\begin{array}{c} \Delta ={\text{var}}_{D}\left[h\left(D\right)/g\left(D\right)\right]. \end{array}$$ (25)

The limiting variance of ${\hat{N}}_{CDS}$, *V*_11_ from Eq (24), is of primary interest. It can be expanded by elementary operations as

$$\begin{array}{c} \text{var}\left({\hat{N}}_{CDS}\right)=V_{11}=\text{var}\left({\hat{N}}_{known\theta }\right)F \end{array}$$ (26)

where $\text{var}({\hat{N}}_{known\theta })$ is the variance of $\hat{N}$ when ***θ*** is known, as defined by Eq (12), and

$$\begin{array}{c} F=1+{\bar{h}}^{T}\Delta^{-1}\bar{h}{\bar{g}}^{-2}{\left(1-P\bar{g}\right)}^{-1} \end{array}$$ (27)

is a penalty term attributable to ***θ*** requiring estimation. The penalty *F* is always 1 or greater, because Δ is a variance-covariance matrix, and so is positive semi-definite.

The coefficient of variance (CV) of ${\hat{N}}_{CDS}$ follows directly, noting that $E\left[n\right]=NP\bar{g}$:

$$\begin{array}{ccc} CV^{2} & = & \text{var}\left({\hat{N}}_{CDS}\right)/N^{2}=FNP^{-1}{\bar{g}}^{-1}\left(1-P\bar{g}\right)/N^{2} \end{array}$$ (28)

$$\begin{array}{ccc} & = & F\left(1-E[n]/N\right)/E\left[n\right] \end{array}$$ (29)

### 2.3 Numerical values of the asymptotic variance for selected models

Values of Δ are obtained by rewriting Eq (25) as

$$\begin{array}{c} \Delta =\frac{w^{-1}\int_{0}^{w}h\left(u\right)h{\left(u\right)}^{T}g{\left(u\right)}^{-1}du}{\bar{g}}-\frac{\bar{h}{\bar{h}}^{T}}{{\bar{g}}^{2}} \end{array}$$ (30)

and calculating by quadrature using the integrate function in the R Statistical Environment [28]. Table 2 shows values of *F* numerically calculated for various hazard rate models. The parameter *θ*_2_ determines the shape of the detection curve, with 1.1 giving a very narrow shoulder (i.e. steeply declining for small distances) and 3 giving a very wide shoulder. Hazard rate detection models for a number of values of *θ*_2_ are illustrated in the next section. The parameter *θ*_1_ is calculated numerically to give the specified $\bar{g}$ in each row.

**Table 2:** Asymptotic penalty (*F*) for the hazard rate model for selected values of the coverage rate *P*, the shape parameter (*θ*_2_) and the mean detection rate $\bar{g}$. The largest distance for which detection is attempted is *w* = 1 in all cases.

*P*	$\bar{g}$	*θ*_2_					
		1.1	1.25	1.5	2	2.5	3
0.1	0.3	6.60	5.28	3.99	2.79	2.25	1.94
0.3	0.3	6.97	5.56	4.19	2.91	2.33	2.00
0.6	0.3	7.63	6.06	4.54	3.12	2.47	2.11
0.9	0.3	8.44	6.68	4.97	3.38	2.65	2.25
0.1	0.6	5.03	4.25	3.43	2.59	2.17	1.92
0.3	0.6	5.62	4.72	3.78	2.82	2.34	2.05
0.6	0.6	6.92	5.77	4.56	3.33	2.71	2.34
0.9	0.6	9.23	7.63	5.96	4.24	3.38	2.87
0.1	0.9	3.28	2.93	2.54	2.09	1.85	1.70
0.3	0.9	3.85	3.41	2.91	2.36	2.06	1.87
0.6	0.9	5.52	4.82	4.04	3.16	2.69	2.39
0.9	0.9	11.94	10.25	8.35	6.24	5.08	4.35

Table 2 shows that *F* increases as *θ*_2_ decreases, i.e. as the shoulder becomes narrower. For a given $\bar{g}$, *F* decreases as the coverage rate *P* increases. This is because increasing *P* improves the precision of ${\hat{N}}_{CDS}$, but it improves the precision of ${\hat{N}}_{known\theta }$ even faster. For a given *P*, *F* decreases as $\bar{g}$ increases when *θ*_2_ ≤ 2 (narrow shoulder), but increases as $\bar{g}$ increases when *θ*_2_ > 2. A possible reason is that when *θ*_2_ is small and $\bar{g}$ is large, the detection function is near 1 for much of its range but then decreases precipitously. The fact that it remains near 1 for much of the range may mean that $\hat{N}$ is relatively insensitive to $\hat{\theta }$. In contrast, when *θ*_2_ > 2 and $\bar{g}$ is large, the detection function declines more smoothly, so that ${\hat{N}}_{CDS}$ is more sensitive to $\hat{\theta }$.

Values of $\bar{g}$ of 0.1, 0.3 or 0.6, *P* = 0.3 and *θ*_2_ ≥ 1.25 are probably the most representative of studies in practice. *F* varies from 1.9 to 5.6 in this subset of Table 2.

Table 3 shows similar results for half-normal detection models. The values of *F* are generally much closer to 1 than in Table 2, varying from 1.5 to 1.9 in the subset of the table where $\bar{g}\in \left\{0.1,0.3,0.6\right\}$ and *P* = 0.3. *F* increases with *P*, as in Table 2. *F* increases with $\bar{g}$ for fixed *P*, similar to the wide-shouldered results in Table 2 where *θ*_2_ > 2.

**Table 3:** Asymptotic penalty (*F*) for the half-normal model for selected values of the coverage rate *P* and the mean detection rate $\bar{g}$. The largest distance for which detection is attempted is *w* = 1 in all cases.

*P*	$\bar{g}$	Penalty (*F*)
0.1	0.3	1.52
0.3	0.3	1.55
0.6	0.3	1.61
0.9	0.3	1.69
0.1	0.6	1.78
0.3	0.6	1.90
0.6	0.6	2.15
0.9	0.6	2.60
0.1	0.9	2.23
0.3	0.9	2.54
0.6	0.9	3.44
0.9	0.9	6.90

## 3 Simulation study

### 3.1 Design of the simulation study

#### Generation of distances *d*_*i*_ for *i* = 1, …, *N*

Distance data are simulated for abundances *N* such that the expected numbers of detections are *E*[*n*] = 50, 100, 200, …, 1000, and the fraction of the area covered is *P* = 0.1. 10,000 simulations are used in every case.

Distances *d*_*i*_ are generated both with and without overdispersion. In the latter case, *d*_*i*_ are independent *U*(0, 10) random variables. The maximum range of observation is set to *w* = 1, so that objects are only eligible for detection when *d*_*i*_ ≤ 1, and so the probability of any given object falling within the covered area is *P* = 0.1. One implication of this model is that the number of objects *n*(*v*) with distance falling into any given interval of length *v* within [0, 10] is distributed as *n*(*v*)∼*bin*(*N*, *vP*), and hence *E*[*n*(*v*)] = *NvP* and *var*[*n*(*v*)] = *NvP*(1 − *vP*). For example, *N*_*c*_ is a special case of *n*(*v*) with *v* = 1. The assumption of independent uniform distances has been criticised because in practice *n*(*v*) is often observed to be overdispersed, with variance greater than *NvP*(1 − *vP*).

Overdispersed *d*_*i*_ are generated by firstly replacing the *U*(0, 10) distribution by the discrete approximation with probability 0.001 at each of 1000 evenly spaced values between 0 and 10. The probability that *d*_*i*_ falls in any given interval would then be 0.001 in the non-overdispersed case. Overdispersed *d*_*i*_ are assumed to be discrete random variables with the same set of possible values, with probability *ϕ*_*k*_ for value *k* = 1, …, 1000. The vector ***ϕ*** is simulated as coming from a Dirichlet distribution with vector parameter equal to 0.001*α* **1**_**1000**_ where **1**_**1000**_ is a vector containing 1000 values all equal to 1, and *α* is a parameter which controls the variance of ***ϕ***. When *α* → ∞, ***ϕ*** is equal to **1**_**1000**_/1000 with probability 1, resulting in the non-overdispersed case. For 0 < *α* < ∞, ***ϕ*** are random variables each lying between 0 and 1, with $\sum_{k=1}^{1000}\phi_{k=1}$. The parameters ***ϕ*** are generated anew for each simulation, and {*d*_*i*_:*i* = 1, …, *N*} are then generated as independent discrete random variables with probabilities ***ϕ*** at each of the 1000 evenly spaced values between 0 and 10.

The overdispersed *d*_*i*_ have the property that *n*(*v*) is beta-binomial distributed with parameters *N*, *αPv*, and *α*(1 − *Pv*). (This follows from the properties of the Dirichlet-multinomial and beta-binomial distributions—see for example [29].) The expected value of *n*(*v*) is then *NvP* as before, but the variance is inflated to *var*[*n*(*v*)] = *cNvP*(1 − *vP*) where *c* = (*α* + *N*)/(*α* + 1) is an overdispersion factor. Values of *α* corresponding to *c* = 1, 2 and 3 are used.

The above process is a discrete approximation of a Dirichlet process with base distribution given by *U*(0, 10). Dirichlet processes are widely used as prior distributions for distribution functions (e.g. chapter 23 of [30]). This also makes them suitable to simulate overdispersed data, particularly as the resulting *d*_*i*_ have the property that the number of distances falling in any interval is overdispersed to the same degree.

#### Simulation of detection process

**Fig 1:**
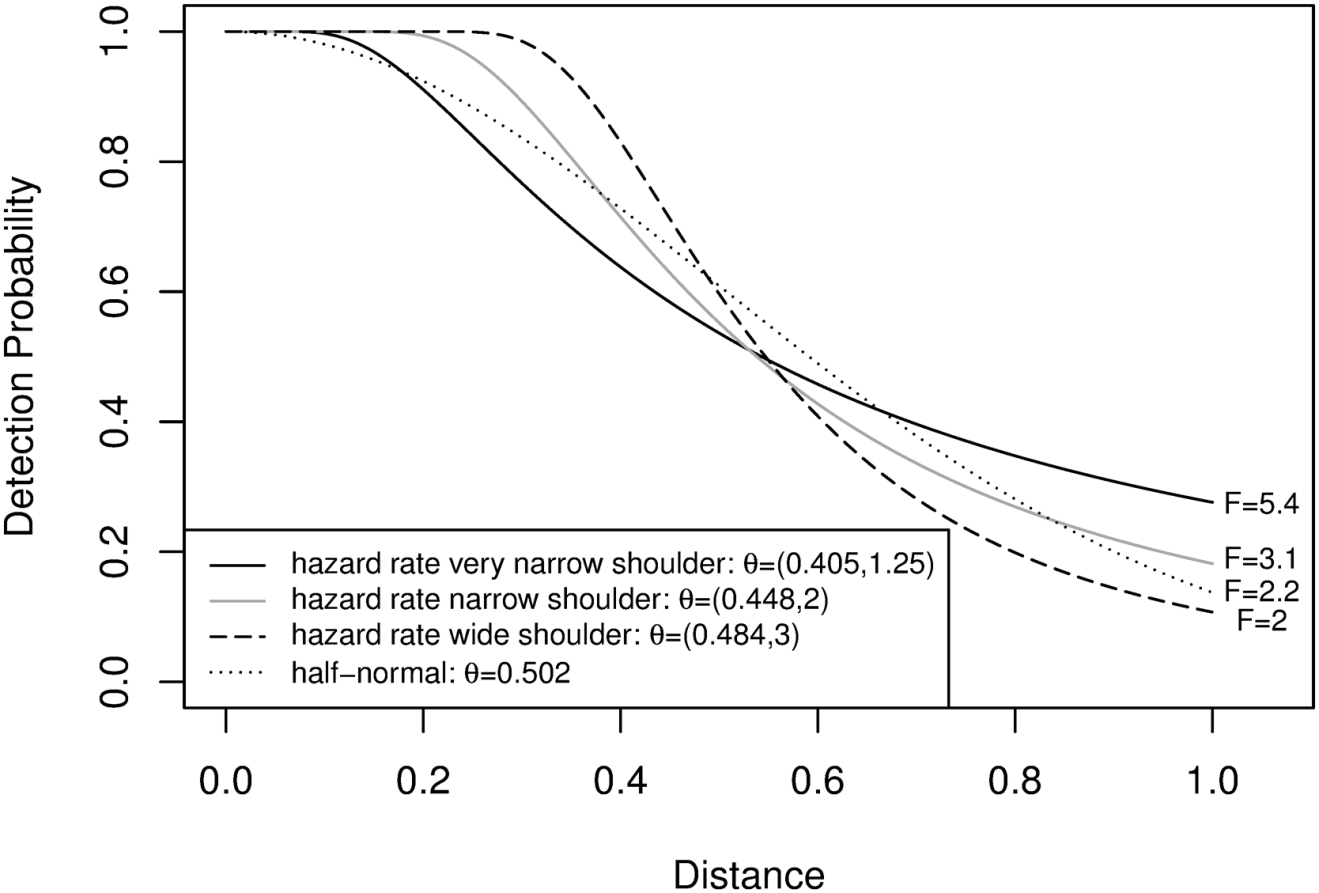
Detection Functions used to Generate Simulated Data. The variance penalty factors *F* from Eq (26) due to unknown ***θ*** are also shown.

Objects are detected with probability *g*(*d*_*i*_; ***θ***) when *d*_*i*_ ≤ *w* = 1 for each *i*, with detection independent across objects. Two families of detection functions are used: the hazard rate and the half-normal. Both are among those proposed in [9]. The two-parameter hazard rate function meets the requirement specified on page 41 of [9] of being a flexible model, giving some robustness to mis-specified detection function; in particular, it allows the shoulder to be narrow or wide. The half-normal detection function is less flexible, but is also often used, and would generally be easier to fit from data as it has only one parameter. Fig 1 shows the 4 particular distance functions used: hazard rate (hr) functions $g\left(d_{i};\theta \right)=1-e^{-{\left(d_{i}/\theta_{1}\right)}^{-\theta_{2}}}$ with ***θ*** = (0.405, 1.25), ***θ*** = (0.448, 2) and ***θ*** = (0.484, 3) corresponding to a very narrow, narrow, and wide shoulder respectively; and a half-normal (hn) function $g\left(d_{i};\theta \right)=1-e^{-{\left(d_{i}/\theta \right)}^{2}/2}$ with *θ* = 0.502. This gives a variety of shapes of the detection function, with all 4 having the same average detection rate of $\bar{g}\left(\theta \right)=0.6$. This means that the number of detections is approximately $n\approx P\bar{g}\left(\theta \right)N=0.06N$. The values of *g*(*w*) for all three functions are at least 0.11, and all but the wide hazard rate function are at least 0.14, roughly in line with the rule of thumb for choice of *w* on page 16 of [9]. The figure also shows the asymptotic penalty *F* due to unknown ***θ*** for each detection function. The penalty ranges from 2 to 5.4.

#### Estimators of abundance

The CDS estimator ${\hat{N}}_{CDS}$ defined in Eq (6) is calculated using the hazard rate model and the half-normal model. The CDS estimator assuming known ***θ***, defined in Eq (11), is also calculated. This last option is of course unrealistic in practice, but is included to show the impact of uncertainty about ***θ***.

An estimate of *N* is also calculated using the strip transect estimator ${\hat{N}}_{ST}=n/P$. This is unbiased under the binomial model *n* ∼ *bin*(*N*, *P*), which incorrectly assumes perfect detection up to distance *w*. The strip transect estimator is also applied to restricted datasets using only those distances up to a range of *w*′ = 0.01, 0.02, …, 1, with ${\hat{N}}_{ST}\left(w^{\prime }\right)=n\left[d\leq w^{\prime }\right]/\left(Pw^{\prime }/w\right)$.

Each simulation corresponds to a single detection function, and a single value of *E*[*n*] and of *c*. 10,000 populations of distances and detections are generated in each simulation. All computations are carried out in the R statistical environment version 3.0.1 [28]. The Distance package [31] is not used because of occasional non-convergence (this would generally not be an issue in practice, but is a problem in a large simulation). The estimator ${\hat{\theta }}_{CML}$ is calculated by maximising *l*_*d*_ from Eq (3) using the optim function, using the Nelder-Mead method for the two-parameter hazard rate function and the Brent method for the one-parameter half-normal function. The complete simulation requires approximately 14 hours on a Macbook Pro with a 2.7GHz Intel Core i7 processor and 16GB of RAM. The code to conduct the simulation and produce the figures and tables is in S1 Code.

The aim of the simulation is to estimate the penalty factor due to unknown detection parameters for finite sample sizes, and to compare the mean squared errors (MSEs) of line transect and strip transect estimators in different scenarios. The focus of the paper is on variances and mean squared errors of abundance estimators, so variance estimators and confidence interval coverage are not reported on.

### 3.2 Simulation results for the asymptotic penalty

**Fig 2:**
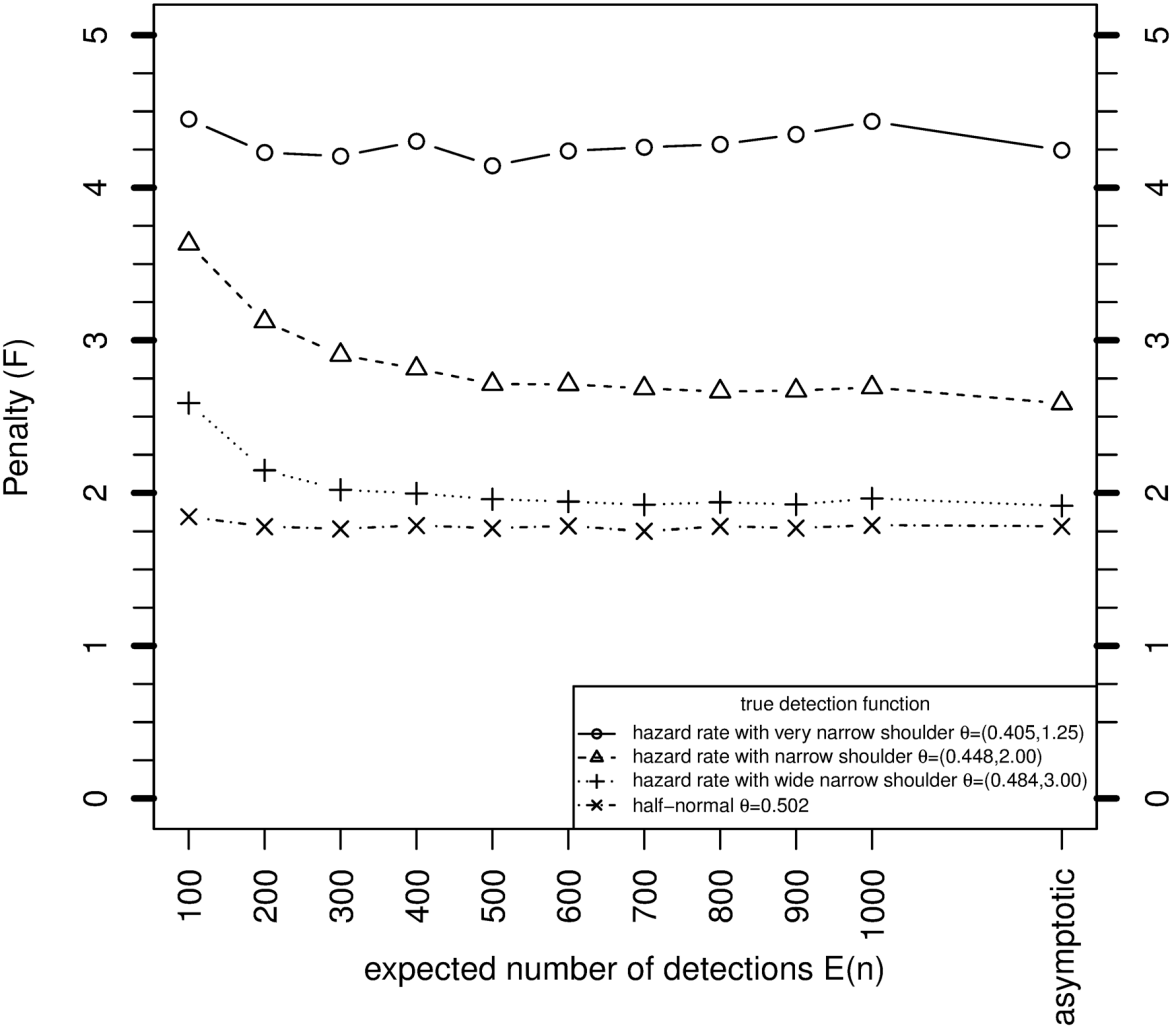
Variance of MLE of *N* when *θ* is unknown relative to when it is known, for various sample sizes based on simulation, and asymptotically from Eq (26), where *P* = 0.1, *c* = 1 and $\bar{g}=0.6$.

**Fig 3:**
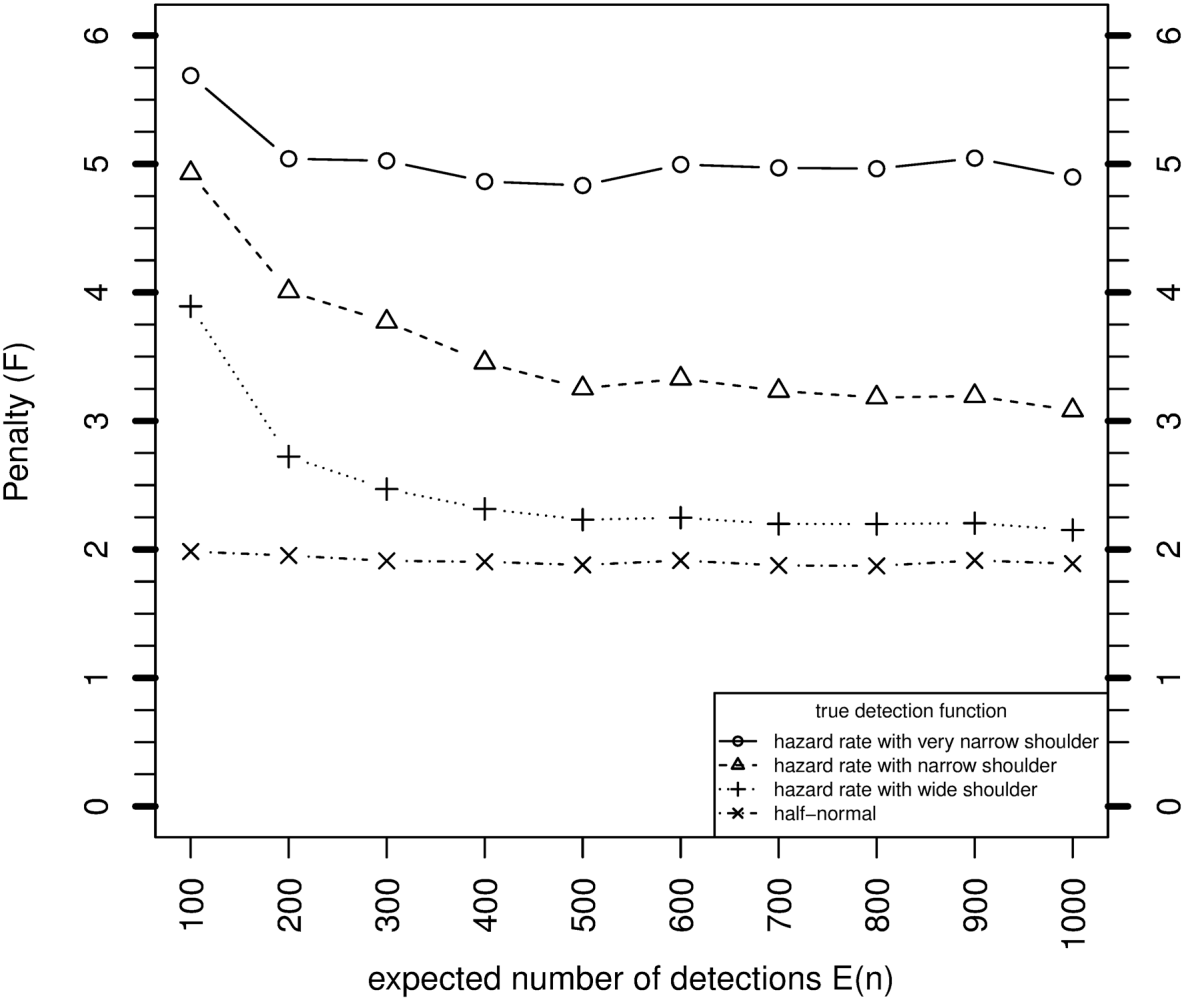
Variance of MLE of *N* when *θ* is unknown relative to when it is known, for various sample sizes based on simulation, and asymptotically from Eq (26), where *P* = 0.1, *c* = 2 and $\bar{g}=0.6$.

Fig 2 shows how the simulation estimates of *F* converge to the asymptotic values as *n* and *N* increase, for *c* = 1. For the hazard rate with very narrow shoulder and the halfnormal model, the asymptotic approximation is good even for *E*[*n*] = 100. For the other two models, the small sample penalties are higher than the asymptotic value for *E*[*n*] = 100, but converge to the asymptote as *E*[*n*] increases. The results provide a computational confirmation of the derivation of *F* in Section 3. Fig 3 shows the simulation estimates of *F* when the overdispersion parameter *c* is 2. No asymptotic result for *F* is available in this case. The values of *F* are 0.1–0.7 higher than when *c* = 1.

### 3.3 Simulation MSEs of CDS and strip transect estimators

**Fig 4:**
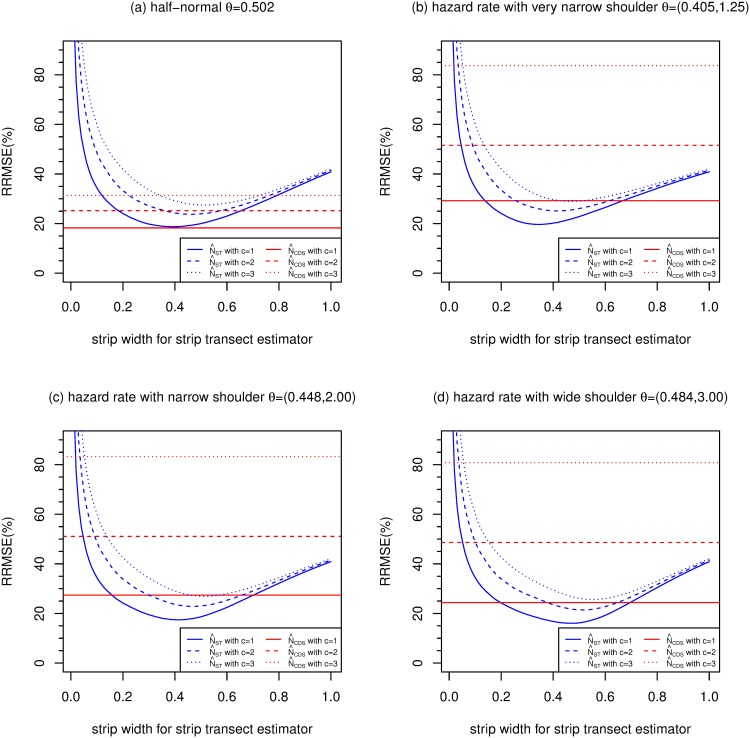
Relative root mean squared errors (RRMSE) (%) of CDS and strip transect estimators estimators (strip widths ranging from 0 to 1) of *N*, for 4 detection functions, with *E*[*n*] = 50, *P* = 0.1 and $\bar{g}=0.6$.

Fig 4 compares the simulation MSEs of ${\hat{N}}_{CDS}$, and ${\hat{N}}_{ST}$ with varying strip width, when *E*[*n*] = 50. The 3 horizontal red lines in each plot are the MSEs of ${\hat{N}}_{CDS}$, which make use of all detections up to distance *w* = 1. The 3 lines are for overdispersion values of *c* = 1, 2 and 3. The 3 blue curves show the MSEs of ${\hat{N}}_{ST}$ for *c* = 1, 2 and 3, with strip widths of 0.01, 0.02, …, 1. Fig 4(a) shows MSEs for the half-normal detection function. Fig 4(b), 4(c) and 4(d) are results for the hazard rate models with very narrow, narrow and wide shoulders, respectively.

The format of Fig 4 is loosely based on Figure 1 in [15]. [15] compared CDS and strip transect estimators for the half-normal and negative exponential detection functions (both one parameter families) for *n* = 40, 60 and 100, and *c* = 1, 2 and 3. Here we extend these results to the two-parameter hazard rate function, and we simulate over-dispersed distances corresponding to *c* = 1, 2 and 3, rather than using the approximate formula in [15] to convert results when *c* = 1 to other values of *c*.

**Fig 5:**
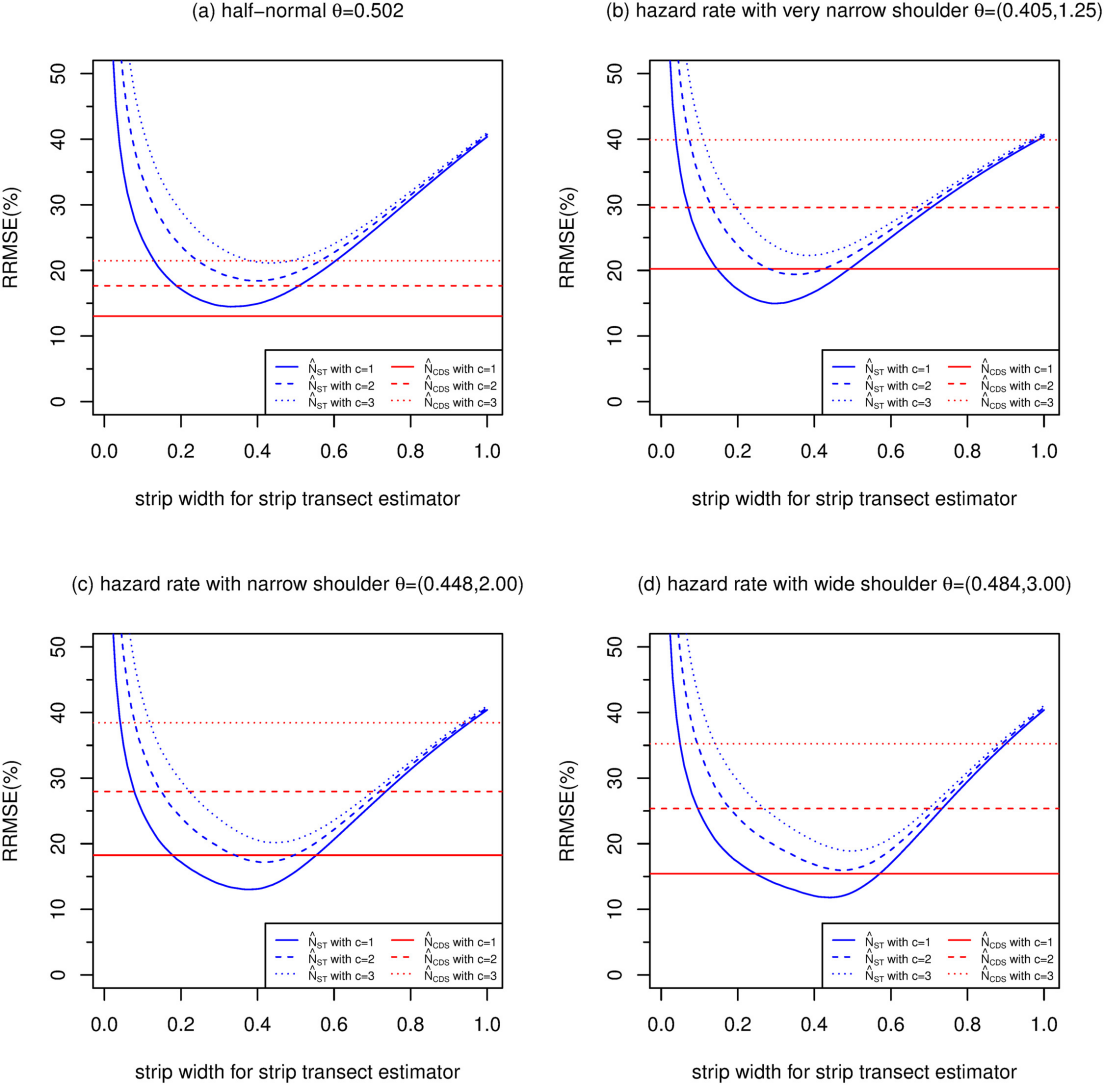
Relative root mean squared errors (RRMSE) (%) of CDS and strip transect estimators estimators (strip widths ranging from 0 to 1) of *N*, for 4 detection functions, with *E*[*n*] = 100, *P* = 0.1 and $\bar{g}=0.6$.

**Fig 6:**
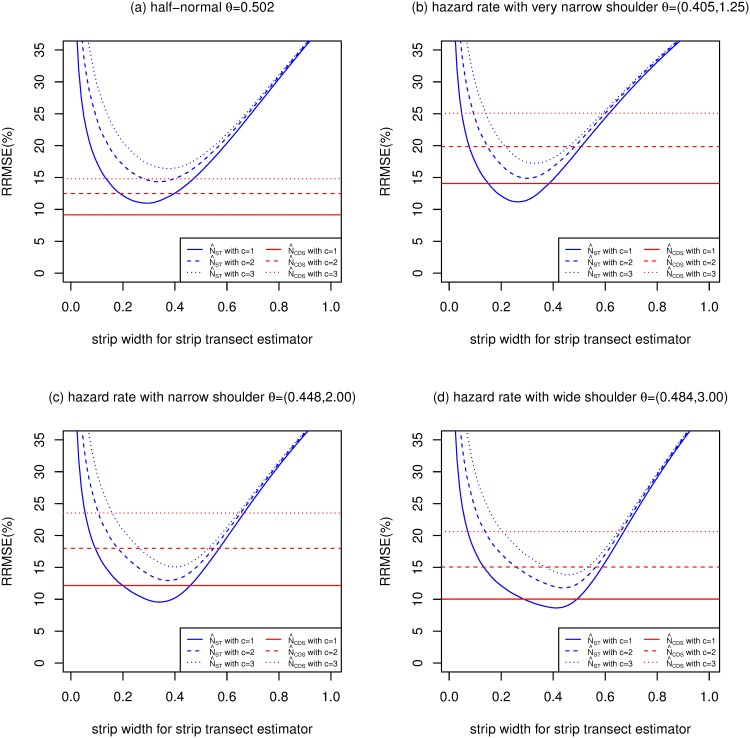
Relative root mean squared errors (RRMSE) (%) of CDS and strip transect estimators estimators (strip widths ranging from 0 to 1) of *N*, for 4 detection functions, with *E*[*n*] = 200, *P* = 0.1 and $\bar{g}=0.6$.

**Fig 7:**
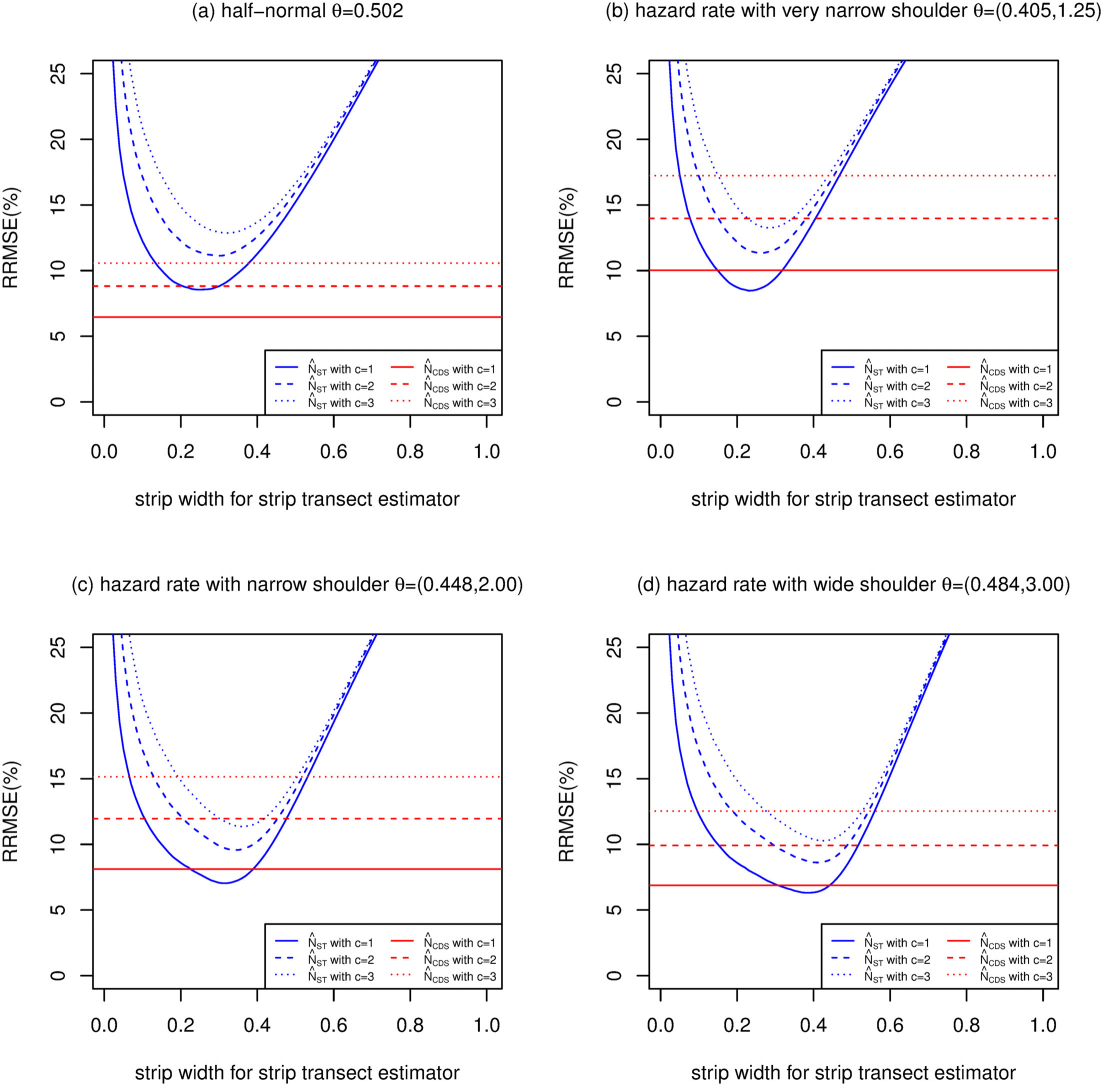
Relative root mean squared errors (RRMSE) (%) of CDS and strip transect estimators estimators (strip widths ranging from 0 to 1) of *N*, for 4 detection functions, with *E*[*n*] = 400, *P* = 0.1 and $\bar{g}=0.6$.

Figs 5, 6 and 7 are of the same format as Fig 4 and show results when *E*[*n*] is 100, 200 and 400 respectively.

For the half-normal function, Figs 4–7 replicate the finding of [15] that CDS dominates strip transect estimators, with the former having lower MSE for almost all values of *c*, *E*[*n*] and strip width.

For the hazard rate function, the picture is quite different, presumably because of the larger value of the penalty (*F*) due to unknown detection parameters for this model. [15] argue that *c* = 2 is the most practically relevant scenario out of *c* = 1, 2 and 3, so we concentrate on this case. For this value of *c*, and for the narrow shoulder detection function (panel (c) of each figure), ${\hat{N}}_{ST}$ has lower MSE than ${\hat{N}}_{CDS}$ when the strip width is 0.1 and above (when *E*[*n*] = 50), between 0.15 and 0.71 (when *E*[*n*] = 100), 0.19 and 0.54 (when *E*[*n*] = 200) and 0.21 and 0.45 (when *E*[*n*] = 400). The optimal strip widths are 0.47, 0.42, 0.38 and 0.35 for *E*[*n*] equal to 50, 100, 200 and 400, respectively.

${\hat{N}}_{CDS}$ performs better relative to ${\hat{N}}_{ST}$ than the above when the hazard rate function has a wide shoulder, and worse than the above when the shoulder is very narrow.

The MSEs of both ${\hat{N}}_{CDS}$ and ${\hat{N}}_{ST}$ increase as *c* increases, particularly ${\hat{N}}_{ST}$. So when *c* increases, $MSE[{\hat{N}}_{CDS}]/MSE[{\hat{N}}_{ST}]$ decreases slightly.

Simulations were also carried out with values of *P* other than 0.1. Results for *P* equal to 0.2, 0.25, 0.3 and 0.5, with *E*[*n*] = 100, are in S1, S2, S3 and S4 Figs, respectively. A comparison of these figures to Fig 4 shows that mean squared errors decrease slightly as *P* increases. The relative performance of the different methods for the various *c* and *w* is insensitive to *P*.

## 4 Conclusions

To achieve a given coefficient of variation *CV* for the line transect estimator, the required expected sample size is

$$\begin{array}{c} E\left[n\right]={\left(N^{-1}+CV^{2}F^{-1}\right)}^{-1} \end{array}$$ (31)

(follows from rearranging Eq (29)). When *n* is much smaller than *N*, this simplifies to

$$\begin{array}{c} E\left[n\right]=F/CV^{2}. \end{array}$$ (32)

The factor *F* is the inflation due to unknown detection function parameters. Asymptotic values of *F* are made available here for the first time (see Tables 2 and 3), albeit under a strong simplifying assumption that counts are binomially distributed. The asymptotic values of *F* for typical hazard rate models are between 2 and 6. Simulation confirms the accuracy of the asymptotic result when there is no overdispersion, and shows that *F* is larger in the presence of overdispersion. The R package distance.sample.size[32] calculates *F* and required sample size using the methods described in the current paper.

Note that the inflation factor *F* does not apply if *g* is modelled using mark-recapture data as suggested by [14].

The results on *F* help to explain the relative mean squared errors of strip and CDS estimators in the simulation. When the number of detections *n* is sufficiently large (e.g. 400), CDS outperforms strip transect estimation unless the strip width is moderately close to its optimal value (within about ± 25%). This is because variances of estimated abundances are then relatively small, so that bias considerations are paramount. For smaller *n*, the variance of the CDS estimator becomes more dominant, partly due to the penalty *F*. As a result, when the overdispersion factor is 2 and the hazard rate model applies, we find that strip transects give lower mean squared error (MSE) than CDS when:

- *n* = 50 and the strip width is 0.1 or higher (with an optimal width of about 0.4), where a width of 1 corresponds to a typical detection range for distance sampling;
- *n* = 100 and the strip width is between 0.2 and 0.7, i.e. within about ± 75% of its optimal value of 0.42; or
- *n* = 200 and the strip width is within about 50% of its optimal value.

A Dirichlet process approach was able to generate non-uniform detections resulting in overdispersion factors of 1, 2 and 3. This approach has not been used in simulations in statistical ecology to the author’s knowledge. It would be applicable to any simulation where overdispersion or robustness relative to an assumed spatial model is of interest.

The simulation settings are more favourable to CDS than to strip transect estimators. In particular, it is assumed that the functional form used in CDS estimation matches the true detection function. This assumption will never be perfectly justified in reality, and its failure will impact on the performance of CDS estimators to some extent. Model selection or model averaging are often used to try to identify the correct model, but uncertainty about the model form will inflate variances and potentially lead to some bias as well. In contrast, strip transect estimators do not require a specification of the detection function. Of course, the properties of strip transect estimators depend on the mean detection rate in the strip, but beyond that there is no particular impact of one functional form rather than another. The simulations with *c* = 1 are also favourable to both CDS and strip transects as they mean that distances are independent and uniformly distributed. This is relaxed to some extent by the overdispersed simulations with *c* equal to 2 and 3, however the Dirichlet process used is still centred on the uniform distribution. When there is a more severe departure from uniformity, CDS estimators will be biased, and strip transect estimators will also be affected to some degree.

The choice of methodology for assessing abundance, as well as the determination of required sample size, should be informed by consideration of all relevant biases and by the likely achievable precision. The results in this paper will help in this process, by providing a sample size formula reflecting the penalty due to unknown detection parameters, theoretical and simulation results on the size of this penalty, and a comparison of the mean squared errors of strip and line transect estimators in a wide-ranging simulation.

## Appendix: Proof of Theorem 1

Applying Eq (15), we approximate *l* = log(*L*) by

$$\begin{array}{ccc} l & \approx & N\log\left(N\right)-\left(N-n\right)\log\left(N-n\right)-n\log\left(n\right)+\left(N-n\right)\log\left(1-P\bar{g}\left(\theta \right)\right)\\ & & +n\log\left(P\right)-n\log\left(w\right)+\sum_{i=1}^{n}\log g\left(d_{i};\theta \right) \end{array}$$ (33)

The next step is to differentiate *l* to obtain the score function:

$$\begin{array}{ccc} \frac{\partial l}{\partial N} & = & N\cdot N^{-1}+1\cdot \log\left(N\right)-\left(N-n\right)\cdot {\left(N-n\right)}^{-1}-\log\left(N-n\right)+\log\left(1-P\bar{g}\left(\theta \right)\right) \end{array}$$ (34)

$$\begin{array}{ccc} & = & \log\left(N\right)-\log\left(N-n\right)+\log\left(1-P\bar{g}\left(\theta \right)\right) \end{array}$$ (35)

$$\begin{array}{ccc} \frac{\partial l}{\partial \theta } & = & \sum_{i=1}^{n}g{\left(d_{i};\theta \right)}^{-1}h\left(d_{i};\theta \right)-\left(N-n\right){\left(1-P\bar{g}\left(\theta \right)\right)}^{-1}P\bar{h}\left(\theta \right) \end{array}$$ (36)

The MLE is obtained by setting Eqs (35) and (36) to 0. Firstly, set Eq (35) to 0 and exponentiate both sides:

$$\begin{array}{ccc} 1 & = & \hat{N}{\left(\hat{N}-n\right)}^{-1}\left(1-P\bar{g}\left(\hat{\theta }\right)\right) \end{array}$$ (37)

which leads directly to

$$\begin{array}{c} \hat{N}=n/\left\{P\bar{g}\left(\hat{\theta }\right)\right\}. \end{array}$$ (38)

Setting Eq (36) to 0, and then substituting for $\hat{N}$ from Eq (38) gives an estimating equation for ***θ***:

$$\begin{array}{ccc} 0 & = & \sum_{i=1}^{n}g{\left(d_{i};\theta \right)}^{-1}h\left(d_{i};\theta \right)-\left(\hat{N}-n\right){\left(1-P\bar{g}\left(\theta \right)\right)}^{-1}P\bar{h}\left(\theta \right) \end{array}$$ (39)

$$\begin{array}{ccc} & = & \sum_{i=1}^{n}g{\left(d_{i};\theta \right)}^{-1}h\left(d_{i};\theta \right)-\left(nP^{-1}\bar{g}{\left(\theta \right)}^{-1}-n\right){\left(1-P\bar{g}\left(\theta \right)\right)}^{-1}P\bar{h}\left(\theta \right) \end{array}$$ (40)

$$\begin{array}{ccc} & = & \sum_{i=1}^{n}g{\left(d_{i};\theta \right)}^{-1}h\left(d_{i};\theta \right)-nP^{-1}\bar{g}{\left(\theta \right)}^{-1}\left(1-P\bar{g}\left(\theta \right)\right){\left(1-P\bar{g}\left(\theta \right)\right)}^{-1}P\bar{h}\left(\theta \right) \end{array}$$ (41)

$$\begin{array}{ccc} & = & \sum_{i=1}^{n}g{\left(d_{i};\theta \right)}^{-1}h\left(d_{i};\theta \right)-n\bar{g}{\left(\theta \right)}^{-1}\bar{h}\left(\theta \right) \end{array}$$ (42)

Results Eqs (38) and (42) give the Theorem result on the maximum likelihood estimators of *N* and ***θ***.

The Fisher Information is given by the variance of the score vector, the elements of which are given by Eqs (35) and (36). It can be written as

$$\begin{array}{c} I\left(N,\theta \right)=\left[\begin{array}{cc} I_{NN} & I_{N\theta }^{T}\\ I_{N\theta } & I_{\theta \theta } \end{array}\right]=\left[\begin{array}{cc} \text{var}\left(\partial l/\partial N\right) & \text{cov}{\left(\partial l/\partial N,\partial l/\partial \theta \right)}^{T}\\ \text{cov}\left(\partial l/\partial N,\partial l/\partial \theta \right) & \text{var}\left(\partial l/\partial \theta \right) \end{array}\right]. \end{array}$$ (43)

The block elements of I are easily derived. Some preliminary notes:

1. Let *D* be a random variable with density $g\left(d;\theta \right)/\int_{0}^{w}g\left(u;\theta \right)du$ for 0 ≤ *d* ≤ *w*.
2. The distances *d*_*i*_ follow the same distribution as *D*, conditional on *n*.
3. $E(g{\left(d_{i};\theta \right)}^{-1}h(d_{i};\theta )|n)=\int_{0}^{w}g{\left(d;\theta \right)}^{-1}h(d;\theta )g(d;\theta )dd/\int_{0}^{w}g\left(u;\theta \right)du=\bar{h}(\theta )/\bar{g}(\theta ).$

For the remainder of the proof, I will write *g*(*u*), ***h***(***u***), $\bar{g}$ and $\bar{h}$ for readability, rather than *g*(*u*; ***θ***) etc. Using (a), (b) and (c), we obtain:

$$\begin{array}{ccc} I_{\theta \theta } & = & \text{var}\left(\partial l/\partial \theta \right)=\text{E}\left\{\text{var}\left(\partial l/\partial \theta |n\right)\right\}+\text{var}\left\{\text{E}\left(\partial l/\partial \theta |n\right)\right\} \end{array}$$ (44)

$$\begin{array}{ccc} & = & \text{E}\left(n\text{var}\left(h\left(D\right)/g\left(D\right)\right)\right)+\text{var}\left\{n\bar{h}{\bar{g}}^{-1}-\left(N-n\right){\left(1-P\bar{g}\right)}^{-1}P\bar{h}\right\} \end{array}$$ (45)

$$\begin{array}{ccc} & = & E\left[n\right]\text{var}\left(h(D)/g(D)\right)+\text{var}\left\{n\bar{h}{\bar{g}}^{-1}{\left(1-P\bar{g}\right)}^{-1}\left(1-P\bar{g}+P\bar{g}\right)+\text{const.}\right\} \end{array}$$ (46)

$$\begin{array}{ccc} & = & E\left[n\right]\text{var}\left(h(D)/g(D)\right)+\text{var}\left\{n\bar{h}{\bar{g}}^{-1}{\left(1-P\bar{g}\right)}^{-1}\right\} \end{array}$$ (47)

$$\begin{array}{ccc} & = & NP\bar{g}\text{var}\left(h(D)/g(D)\right)+\text{var}\left(n\right)\bar{h}{\bar{h}}^{T}{\bar{g}}^{-2}{\left(1-P\bar{g}\right)}^{-2} \end{array}$$ (48)

$$\begin{array}{ccc} & = & NP\bar{g}\text{var}\left(h(D)/g(D)\right)+NP\bar{g}\left(1-P\bar{g}\right)\bar{h}{\bar{h}}^{T}{\bar{g}}^{-2}{\left(1-P\bar{g}\right)}^{-2} \end{array}$$ (49)

$$\begin{array}{ccc} & = & NP\bar{g}\text{var}\left(h(D)/g(D)\right)+NP\bar{h}{\bar{h}}^{T}{\bar{g}}^{-1}{\left(1-P\bar{g}\right)}^{-1} \end{array}$$ (50)

$$\begin{array}{ccc} I_{N\theta } & = & \text{cov}\left(\partial l/\partial N,\partial l/\partial \theta \right) \end{array}$$ (51)

$$\begin{array}{ccc} & = & \text{E}\text{cov}\left(\partial l/\partial N,\partial l/\partial \theta |n\right)+\text{cov}\left\{\text{E}\left(\partial l/\partial N|n\right),\text{E}\left(\partial l/\partial \theta |n\right)\right\} \end{array}$$ (52)

$$\begin{array}{ccc} & = & 0-\text{cov}\left\{\log\left(N-n\right),n{\bar{g}}^{-1}\bar{h}+n{\left(1-\bar{g}\right)}^{-1}\bar{h}\right\} \end{array}$$ (53)

$$\begin{array}{ccc} & = & -{\bar{g}}^{-1}{\left(1-\bar{g}\right)}^{-1}\bar{h}\text{cov}\left\{\log\left(N-n\right),n\right\} \end{array}$$ (54)

As *N* → ∞ $n\overset{p}{\to }NP\bar{g}$, so the right hand side of Eq (54) can be approximated using a first order Taylor Series of *n* about $E\left[n\right]=NP\bar{g}$:

$$\begin{array}{ccc} I_{N\theta } & \approx & -{\bar{g}}^{-1}{\left(1-P\bar{g}\right)}^{-1}P\bar{h}\cdot \\ & & \text{cov}\left\{\log\left(N-NP\bar{g}\right)-{\left(N-NP\bar{g}\right)}^{-1}\left(n-NP\bar{g}\right),n\right\} \end{array}$$ (55)

$$\begin{array}{ccc} & = & {\bar{g}}^{-1}{\left(1-P\bar{g}\right)}^{-1}\bar{h}N^{-1}{\left(1-P\bar{g}\right)}^{-1}\text{var}\left(n\right) \end{array}$$ (56)

$$\begin{array}{ccc} & = & {\bar{g}}^{-1}{\left(1-P\bar{g}\right)}^{-2}\bar{h}N^{-1}NP\bar{g}\left(1-NP\bar{g}\right) \end{array}$$ (57)

$$\begin{array}{ccc} & = & {\left(1-P\bar{g}\right)}^{-1}P\bar{h} \end{array}$$ (58)

The top-left element of I, $I_{NN}$, can also be approximated by a first order Taylor Series about $n=N\bar{g}$:

$$\begin{array}{ccc} I_{NN} & = & \text{var}\left(\partial l/\partial N\right)=\text{var}\left(\log\left(N-n\right)\right) \end{array}$$ (59)

$$\begin{array}{ccc} & \approx & \text{var}\left\{-\log{\left(N-NP\bar{g}\right)+\left(N-NP\bar{g}\right)}^{-1}\left(n-NP\bar{g}\right)\right\} \end{array}$$ (60)

$$\begin{array}{ccc} & = & N^{-2}{\left(1-P\bar{g}\right)}^{-2}\text{var}\left(n\right) \end{array}$$ (61)

$$\begin{array}{ccc} & = & N^{-2}{\left(1-P\bar{g}\right)}^{-2}NP\bar{g}\left(1-P\bar{g}\right) \end{array}$$ (62)

$$\begin{array}{ccc} & = & N^{-1}{\left(1-P\bar{g}\right)}^{-1}P\bar{g} \end{array}$$ (63)

## Supporting Information

S1 Fig Simulation results for P = 0.2 with E[n] = 100. (EPS)

S2 Fig Simulation results for P = 0.25 with E[n] = 100. (EPS)

S3 Fig Simulation results for P = 0.3 with E[n] = 100. (EPS)

S4 Fig Simulation results for P = 0.5 with E[n] = 100. (EPS)

S1 Code R program used to produce simulation results. (R)
